# Letter to the Editor: The Case for Publicly Funded Headache Surgery in Germany

**DOI:** 10.1016/j.jpra.2021.09.001

**Published:** 2021-09-14

**Authors:** Leonard Knoedler, Christian Chartier, Hassan ElHawary, Andreas Kehrer, Thomas Muehlberger

**Affiliations:** a Department of Plastic, Hand and Reconstructive Surgery, University Hospital Regensburg, Regensburg, Germany; b McGill University Faculty of Medicine, Montreal, Canada; c Division of Plastic and Reconstructive Surgery, McGill University Health Center, Montreal, Canada; d Department of Plastic Surgery and Hand Surgery, DRK-Kliniken Berlin Westend, Humboldt University Berlin, Berlin, Germany; e Migraine Surgery Centre, Harley Street, London, W1G 9PF, United Kingdom

**Keywords:** Headache surgery, Migraine surgery, Migraine, Headache

## Abstract

Headache surgery has become a considerable therapeutic option in headache treatment and is of rising interest in the German medical sector. This viewpoint outlines the need for reimbursement of headache surgery in the German healthcare system and demonstrates its cost-effectiveness. Using state-of-the-art patient selection algorithms, the authors found headache surgery to be cost-effective within 7.2 to 6.3 years. Of note, the approach presented is not limited to the German healthcare system.

*Dear Sir,*

Every tenth German is living with migraine headache.^1^ In this context, Circa 10% of the German population is facing a three to sevenfold increased risk of mood disorders and suicide, and approximately twofold higher probability of population is suffering from myocardial infarctions and apoplexies.^2^^,^^3^

Every day, approximately one million Germans are impaired by migraine episodes, with nearly 60,000 abortive triptans doses taken daily.^4^ Additionally, more than 100,000 certificates of incapacity are issued by doctors to German employers every day. The average annual cost attributable to migraine headache is between $1,820 and $2,100 (direct cost $1,070 vs indirect cost $1,030) for per patient.^5^^,^^6^

The classification of migraine headache as a neurological disorder is supported by the peer-reviewed literature.^7^^,^^8^ However, there is increasing recent evidence that identifies peripheral nerve compression as a plausible culprit in a subset of headache patients.^9^ In these cases, surgical decompression of the supraorbital/supratrochlear, greater occipital, lesser occipital, zygomaticotemporal, or auriculotemporal nerves provides reliable relief of symptoms.^10^ Despite a list of plus points, such as positive surgical outcomes in adequately screened patients, long-term cost-effectiveness, and endorsement by the American Society of Plastic Surgeons (ASPS), headache surgery is not generally reimbursed by German public health insurance to the point that it is even controversially discussed.^9^^,^^11^^,^^12^

Total surgical costs of an index surgical decompression of the frontal and greater occipital trigger sites amount to $8,907 (T. Muehlberger, personal communication, July 8, 2021). Botulinum Toxin A injections cost an additional $547 to $1,094. According to these figures, we estimate the upper bound cost of headache surgery performed in Germany to be $10,002 excluding follow-up costs. According to findings reported by Faber et al., 28.1% (n= 25) of patients who underwent surgery had complete elimination of symptoms and spent negligible sums on headache treatment after surgery (number needed to treat [NNT]= 3.6).^13^ In consequence, headache surgeons must operate on 3.6 patients (totaling $30,006) for one patient to achieve complete remission. Using a more advanced patient selection algorithm developed by Gfrerer et al., yielding a 96% Mean Migraine Headache Index (MHI) reduction (and commensurate health expenditure decrease) across 69% of patients (NNT= 1.5), the breakeven point of each individual's headache surgery occurs at 7.2 years.^9^ In a meta-analysis by Nagori et al., up to 76.4% (n= 879) of patients reported complete relief of headache symptoms after surgery (NNT= 1.3) ^14^. These figures yield a breakeven point at 6.3 years, while a comparable analysis published by Chartier et al. showed headache surgery to be cost-effective in the Canadian healthcare system within 3.6 years.^15^

## Limitations

These points should be interpretated in the light of the following limitations: (1) For the sake of clarity, we assumed each patient achieved their long-term remission immediately after surgery; (2) all costs in this Viewpoint Analytics that are reported in 2021 are in US Dollars (conversions performed based on exchange rates at the time the reference value was published, inflation calculated annually).

In conclusion, robust evidence demonstrates the safety and cost-effectiveness of headache surgery. Thisviewpoint outlines the clear need for its reimbursement in the German healthcare system. Importantly, the authors believe a similar methodology could be applied to comparable healthcare systems in neighboring countries to quicken the worldwide adoption of headache surgery performed on the right patients.

## Declaration of Competing Interest

We have no conflict of interest to disclose.
